# Clustering on longitudinal quality‐of‐life measurements using growth mixture models for clinical prognosis: Implementation on CCTG/AGITG CO.20 trial

**DOI:** 10.1002/cam4.5341

**Published:** 2022-10-24

**Authors:** Jiahui Zhang, Weili Kong, Pingzhao Hu, Derek Jonker, Malcolm Moore, Jolie Ringash, Jeremy Shapiro, John Zalcberg, John Simes, Dongsheng Tu, Chris J. O'Callaghan, Geoffrey Liu, Wei Xu

**Affiliations:** ^1^ Dalla Lana School of Public Health University of Toronto Toronto Ontario Canada; ^2^ Department of Otolaryngology, Head and Neck Surgery, West China Hospital Sichuan University Chengdu Sichuan People's Republic of China; ^3^ Department of Biochemistry Western University London Ontario Canada; ^4^ Ottawa Hospital Research Institute University of Ottawa Ottawa Ontario Canada; ^5^ Department of Medicine and Pharmacology University of Toronto Toronto Ontario Canada; ^6^ Division of Medical Oncology and Hematology Princess Margaret Cancer Centre Toronto Ontario Canada; ^7^ Department of Radiation Oncology The Princess Margaret Cancer Centre Toronto Ontario Canada; ^8^ Cabrini Hospital and Monash University Melbourne Victoria Australia; ^9^ Peter MacCallum Cancer Centre and University of Melbourne; ^10^ NHMRC Clinical Trials Centre University of Sydney Sydney New South Wales Australia; ^11^ Canadian Cancer Trials Group Queen's University Kingston Ontario Canada; ^12^ Temerty Faculty of Medicine University of Toronto Toronto Ontario Canada; ^13^ Department of Biostatistics The Princess Margaret Cancer Centre Toronto Ontario Canada

**Keywords:** cancer treatment, clustering, growth mixture model, quality of life, survival analysis

## Abstract

**Introduction:**

Analyzing longitudinal cancer quality‐of‐life (QoL) measurements and their impact on clinical outcomes may improve our understanding of patient trajectories during systemic therapy. We applied an unsupervised growth mixture modeling (GMM) approach to identify unobserved subpopulations (“patient clusters”) in the CO.20 clinical trial longitudinal QoL data. Classes were then evaluated for differences in clinico‐epidemiologic characteristics and overall survival (OS).

**Methods and Materials:**

In CO.20, 750 chemotherapy‐refractory metastatic colorectal cancer (CRC) patients were randomized to receive Brivanib+Cetuximab (*n* = 376, experimental arm) versus Cetuximab+Placebo (*n* = 374, standard arm) for 16 weeks. EORTC‐QLQ‐C30 QoL summary scores were calculated for each patient at seven time points, and GMM was applied to identify patient clusters (termed “classes”). Log‐rank/Kaplan–Meier and multivariable Cox regression analyses were conducted to analyze the survival performance between classes. Cox analyses were used to explore the relationship between baseline QoL, individual slope, and the quadratic terms from the GMM output with OS.

**Results:**

In univariable analysis, the linear mixed effect model (LMM) identified sex and ECOG Performance Status as strongly associated with the longitudinal QoL score (*p* < 0.01). The patients within each treatment arm were clustered into three distinct QoL‐based classes by GMM, respectively. The three classes identified in the experimental (log‐rank *p*‐value = 0.00058) and in the control arms (*p* < 0.0001) each showed significantly different survival performance. The GMM's baseline, slope, and quadratic terms were each significantly associated with OS (*p* < 0.001).

**Conclusion:**

GMM can be used to analyze longitudinal QoL data in cancer studies, by identifying unobserved subpopulations (patient clusters). As demonstrated by CO.20 data, these classes can have important implications, including clinical prognostication.

## 1 INTRODUCTION

Colorectal cancer (CRC) is the third most commonly diagnosed cancer, and the third deadliest cancer worldwide.^1^, ^2^ The 5‐year overall survival (OS) rates for CRC patients in Canada and Australia are 64% and 69%, respectively.^3^ CRC not only brings physical and psychological discomfort but also greatly affects health‐related quality‐of‐life (henceforth abbreviated as QoL). For decades, improvement in QoL of cancer patients after treatment has been shown to be one indicator of prognosis and survival.^2^, ^4^

The QoL questionnaire designed by the European Organization for Research and Treatment of Cancer (EORTC‐QLQ‐C30) is frequently used in a variety of cancer patient populations.^5^ QoL data can provide supplemental information to help treatment decision‐making.^6^ A number of CRC studies have assessed the association of disease symptoms, treatment toxicities, and QoL, on survival performance. For example, among 2‐ to 11‐year CRC survivors, neuropathy symptoms had significant negative effects on health‐related QoL.^7^ Another systematic review of long‐term CRC survivors stated that although survivors have generally good QoL, they tend to have poorer physical QoL compared with the general population.^8^ Quinten et al suggested that various patterns of symptoms patients experience lead to differential QoL scores and that QoL data can help to predict the prognosis of cancer.^9^ A recent systematic review of CRC survivors found associations between physical activity and longitudinal QoL.^10^ Sehouli et al. also suggested that preoperative QoL data can help to predict postoperative complications for gynecological cancer patients.^11^

The majority of studies on the relationship between health‐related QoL and survival of CRC patients evaluated QoL at a single time‐point, such as trial entry, or time of diagnosis; there is a lack of studies assessing the association between longitudinal QoL and survival.^12^, ^13^, ^14^ Moreover, single time‐point studies have utilized clustering methods to classify either the multiple QoL symptoms or to classify the patients, and sometimes both.^15^, ^16^ Lim et al did examine the association between symptom clusters and clinical factors, where patterns of symptoms are associated with patients that have different clinical characteristics.^17^ Other studies showed that both symptom‐clustering and patient‐clustering (into patient classes) could be used to tailor management strategies.^18^, ^19^ However, single time‐point studies may be suboptimal, compared to using the full longitudinal evolution or trajectories of toxicities/symptoms to help cluster patients into classes.

We are working on a randomized clinical trial CO.20. This trial was conducted by the National Cancer Institute of Canada Clinical Trials Group (NCIC CTG) and the Australasian Gastrointestinal Trials Group (AGITG). The CO.20 Phase III trial randomized 750 chemotherapy‐refractory metastatic CRC patients to receive Brivanib+Cetuximab (*n* = 376, experimental arm) versus Cetuximab+Placebo (*n* = 374, standard arm). QoL data using the EORTC‐QLQ‐C30 patient‐reported questionnaire were collected at baseline and then every 2 weeks until patients left the study (due to disease progression typically, or occasionally due to toxicity or patient preference). Though OS benefit was not observed in this study, progression‐free survival favored the experimental arm. Thus, CO.20 is an optimal study to evaluate the potential clinical utility of longitudinal QoL data, in part to determine whether there are previously unobserved subpopulations of patients in the experimental arm associated with improved OS and to document the clinical characteristics of this subpopulation or class of patients.

To study QoL trajectories, a longitudinal method of evaluating QoL of clustered patients was necessary. A longitudinal study has the advantage over single time‐point data for studying the change patterns of measurements over time. Growth mixture models (GMM) can deal effectively with such time‐related longitudinal data.^20^ GMM is one of the clustering algorithms which is model‐based unsupervised learning. It can help us to identify the latent subgroups of people with longitudinal heterogeneity trajectories. Our main hypothesis is that the subgroups of CRC patients would have different longitudinal QoL which may affect the patients' survival performance. There are few longitudinal studies on the QoL of CRC patients, and there are no studies that utilize clustering of longitudinal QoL using GMM to assess the association of these QoL classes on survival performance of CRC patients. Therefore, the objectives of this study were as follows: (a) to cluster patients on the basis of their longitudinal QoL data by GMM; and (b) to evaluate the association of QoL clusters that were longitudinally generated using GMM with either clinical characteristics or OS.

## 2 METHODS AND MATERIALS

### 2.1 Subjects

Participants of CO.20 were from Australia and Canada and had histologically confirmed, chemotherapy‐refractory, metastatic CRC that was mostly KRAS‐wildtype. The CRC patients were aged 18 years or older and did not have a serious concurrent illness. From the years 2008–2011, CRC patients were 1:1 randomly assigned to the experimental Cetuximab+Brivanib treatment arm or the standard Cetuximab +placebo treatment arm.

### 2.2 QoL Assessment

The Eastern Cooperative Oncology Group (ECOG) Performance Status score describes a patient's level of functioning in terms of their ability to care for themselves, their daily activity, and physical ability. ECOG_PS has five levels. The higher the level, the worse the health condition and the worse the QoL. However, this single value is clinician‐reported, not patient‐reported.

The European Organization for Research and Treatment of Cancer QoL questionnaire (EORTC QLQ‐C30) was used as the patient‐reported QoL measure in this study; this 30‐item questionnaire contains 13 subscales. For all scales/subscales, a linear transformation to standardize the average of the items in the scale was used to obtain a scale score. Instruments were scored according to the standard procedure, which requires 50% of the items to be reported by the patient on each scale. Global summary score on the EORTC‐QLQ‐C30 ranges from 0 to 100. QoL scores at seven different time points (baseline pre‐treatment, week 2, week 4, week 6, week 8, week 12, and week 16) were analyzed; 730 patients completed the QoL questionnaire at least once. One patient did not have baseline QoL and was excluded from further analysis. Over 90% completed the QoL questionnaire at least three times; 83 missing summary scores of QLQ‐C30 were removed as they had either less than 50% of item response in one scale or one missing single‐item symptom score.

### 2.3 Statistical analysis

**FIGURE 1:**
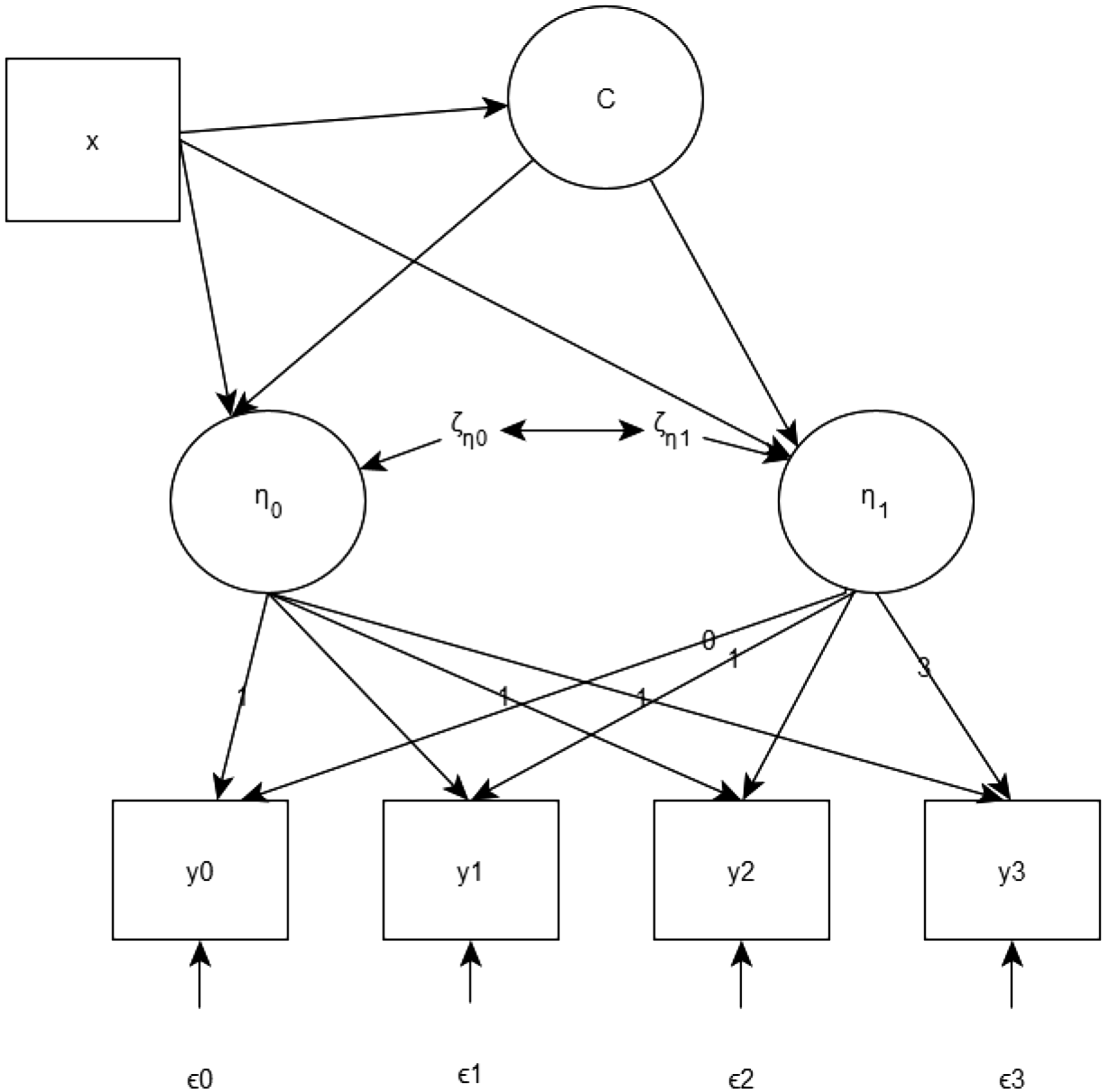
Framework of a linear GMM with predictors at four time points. C is the latent categorical variable; x is the covariate; $\eta_{0}$ and $\eta_{1}$ are latent continuous variables; y_i_ is outcome at time point i; $\zeta_{i}$ and $\epsilon_{i}$ are the residual vectors that are normally distributed

Fisher's exact test was used for comparison on categorical variables and Kruskal–Wallis was used for comparison on continuous variables. A linear mixed effect model (LMM) was used to screen significant variables to establish a GMM. We focused on clustering modeling approaches for longitudinal QoL study. Figure 1 shows an example of the structure of conditional linear GMM (More details can be found in Data S1, ^20^). GMM can help us to identify the latent subgroup of patients based on longitudinal QoLs.

For simplicity, in this study, we assumed that longitudinal QoL data within 16 weeks of observation window were missing at random. We used full information maximum likelihood (FIML) to estimate the model with missing data rather than either excluding missing data or using multiple imputations to generate values for the missing data. FIML is popularly used in Structural Equation Modeling (SEM), multi‐level model or growth models. The FIML estimator maximized the sum of the n‐case‐wise likelihood function with all available raw data.^22^ The likelihood‐ratio chi‐square difference test was conducted when we compared models that had the same number of classes, while Bayesian information criterion (BIC) was used when we compared models with different numbers of classes. To identify the optimal number of classes for each treatment arm, BIC was used in our study.

The method of Kaplan–Meier was used to analyze the landmark OS between different subgroups, starting from the last QoL time‐point (i.e., 16 weeks). Cox regression was applied to test if there was a significant association between baseline QoL, individual slope, and the quadratic term from the GMM output with overall survival for CRC patients. C index, or concordance index, was used to evaluate the prediction ability of the model. Concordance indices estimate the probability that the predicted results are consistent with the actual observed value.^23^ For all comparisons, two‐sided tests were applied with *p* < 0.05 considered the threshold for statistical significance.

## 3 RESULTS

### 3.1 Patient characteristics were well‐balanced between treatment arms

Clinical characteristics were found to be balanced between the two treatment arms.^24^ There were no significant OS differences between the two treatment arms (log‐rank *p* value = 0.12) (Figure S1).

### 3.2 Sex and ECOG performance status were independently robustly associated with QoL in the overall population, adjusted for treatment arm, through LMM

The univariable analysis (UVA) using LMM identified sex and ECOG performance status as being strongly associated with the QoL measurements and was confirmed to be independently significant in multivariable analysis (MVA) after backward elimination for model selection. Therefore, these two variables were incorporated in all adjusted GMMs (Table 1).

**TABLE 1:** Univariable and Multivariable results on QoL using LMM

		Univariable analysis		Multivariable analysis	
		β^a^ (95% CI^b^)	*p* ^c^	β (95% CI)	*p*
Treatment Arm		1.38 (−0.58, 3.35)	0.17		
Age	Per year increase	0.04 (−0.05, 0.13)	0.39		
Sex	Female	Reference^e^		Reference	
	Male	2.01 (1, 3.03)	**<0.001** ^d^	3.99 (2.11, 5.86)	**<0.001**
Primary site	Colon	Reference			
	Colon and Rectum	−0.13 (−1.57, 1.3)	0.86		
	Rectum	−0.76 (−2.87, 1.36)	0.48		
Previous chemo drug classes					
	Yes	Reference			
	No	−2.8 (−7.76, 2.17)	0.27		
Presence of liver metastasis	Yes	Reference			
	No	1.17 (−1.02, 3.35)	0.30		
BMI (kg/m^2^)	Per unit increase	0.09 (−0.09, 0.28)	0.32		
ECOG^f^	0	Reference		Reference	
	1	−8.05 (−10.02, −6.09)	**<0.001**	−8.00 (−9.94, −6.06)	**<0.001**
	2	−16.82 (−20.42, −13.22)	**<0.001**	−16.85 (−20.41, −13.29)	**<0.001**

^a^ β:Regression coefficient estimate.

^b^ 95% confidence interval for each clinical variable by a linear mixed effect model.

^c^ *p*‐value of fixed effect for each clinical variable by a linear mixed effect model using Kenward–Roger approximation.

^d^ *p*‐values less than 0.05 are bolded.

^e^ Reference level for categorical variable in the analysis.

^f^ ECOG, Eastern Cooperative Oncology Group.

Within each treatment arm, the longitudinal global QoL data of the first 16 weeks after study entry was independently and optimally clustered into three classes by GMM. However, the two treatment arms have classes that have different longitudinal patterns of global QoL.

The mean of EORTC‐QLQ‐C30 global summary scores for the two treatment arms is presented in Figure S2. Observed means and individual values for the three‐classes conditional quadratic GMM are shown in Figures S3 and S4. BIC was used to evaluate the optimal number of classes of the GMM.

**FIGURE 2:**
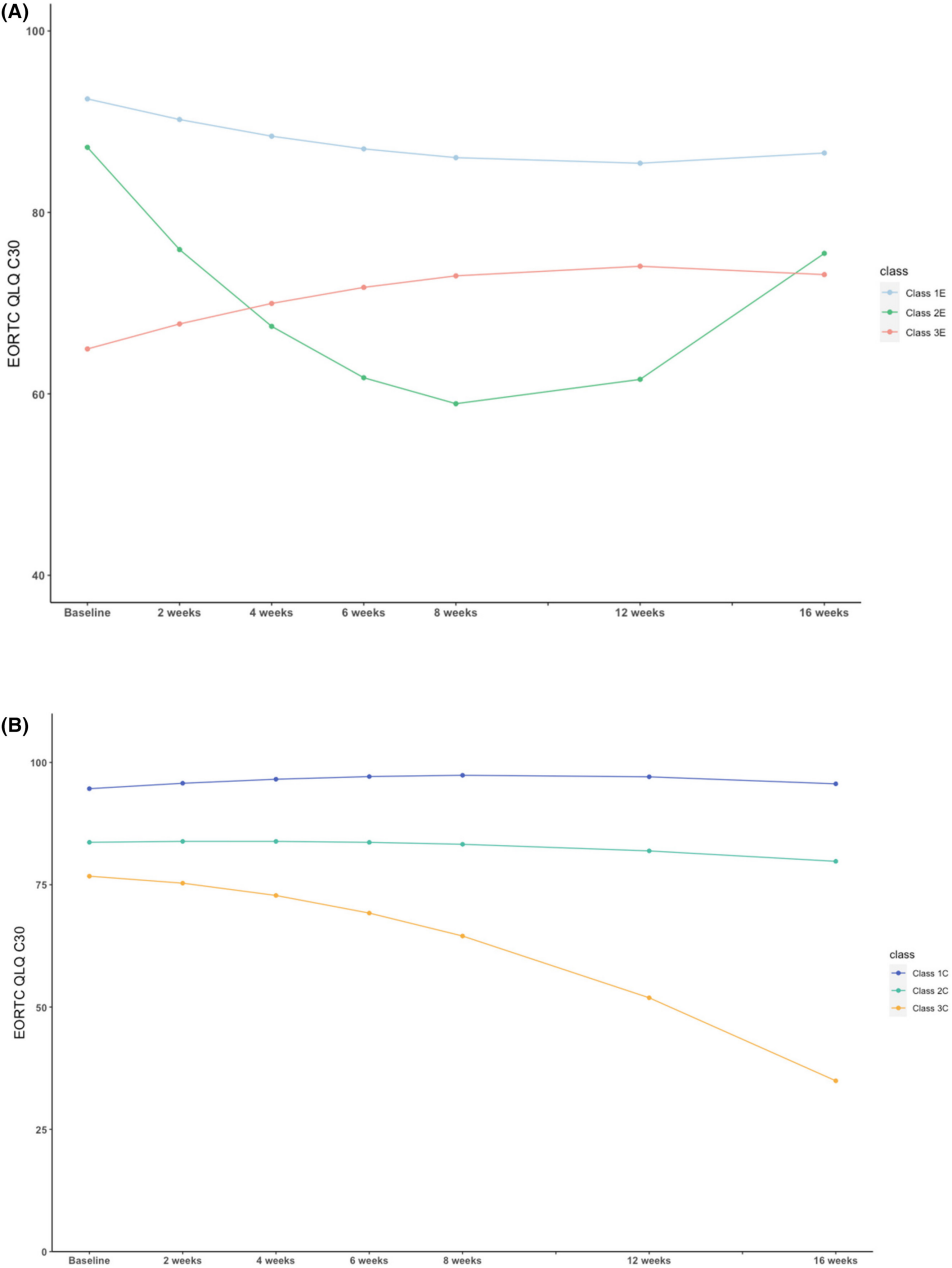
(A) Estimated EORTC QLQ C30 for the three classes of the Cetuximab plus Brivanib treatment arm by GMM. EORTC QLQ C30 is the QoL in our paper. Baseline starts from the randomization.(B) Estimated EORTC QLQ C30 for the Cetuximab plus placebo treatment arm by GMM. EORTC QLQ C30 is the QoL in our paper. Baseline starts from the randomization

For the experimental (E) treatment arm, the BIC was 14,669. The entropy of this GMM was 0.75, which was higher than the acceptable value at 0.7^25^ (Table S1). The proportion for each class in the three‐class conditional GMM was higher than 10%, and thus the number of patients in each class was deemed adequate for analysis.^26^ Due to the performance of BIC, entropy, and proportion of classes, the three‐class was chosen for the GMM models of the experimental arm. GMM clustered the patients based on longitudinal QoL into three classes: Class 1E, Class 2E, and Class 3E. Class 1E had the highest intercept (i.e., best baseline QoL) with a trend toward decreasing QoL. Class 3E had an intermediate intercept with a smooth convex curve. Class 2E had a low (poor baseline QoL) intercept with a non‐significant concave curve (Figure 2A).

For the standard (control, C) treatment arm, the BIC was 14,312, the entropy term was 0.69 (Table S2), and the proportion for each class in the three‐class conditional quadratic GMM was higher than 10%, which was why we chose to divide the patients into three classes for this treatment arm. Three classes were created by GMM:Class 1C, Class 2C, and Class 3C. Class 1C had the highest intercept with a little concave curve. Class 2C had a lower intercept compared with Class 1C and had a non‐significant concave curve. Class 3C also had low intercept and a decreasing trend (Figure 2B). Meanwhile, we used the C‐index to assess the discriminative ability of the model. The results showed that the C‐index values of Gender + ECOG + Class were 0.63 and 0.67 in the two treatment arms, respectively (Table S3).

### 3.3 Different classes identified in GMM clustering analysis of longitudinal global QoL scores (0–16 weeks) within each treatment arm have distinct distributions of clinico‐demographic variables

We compared the clinical characteristics of the new classes of the two treatment arms, respectively. Table 2 shows the clinical variables in each class resulting from the GMM for the experimental treatment arm. There were significant class differences in the following variables: sex and ECOG performance status ($p_{\mathrm{sex}}=0.001;p_{\text{ECOG}}=0.0012$). CRC patients in Class 3E had relatively more females than males, which is noteworthy since there were many more males than females in the total population. Class 1E had the largest number of patients.

**TABLE 2:** Differences in patient subgroups for clinical variables in the Cetuximab plus Brivanib treatment arm

Covariate	Full Sample	Cluster 1E	Cluster 2E	Cluster 3E	*p*‐value^a^
	(*n* = 362)	(*n* = 256)	(*n* = 57)	(*n* = 49)	
Age					0.82
Median (Min, Max^b^)	63.7 (27.1, 83.3)	63.9 (27.1, 83.3)	65 (35.7, 77.4)	63.4 (43.1, 78.4)	
Sex					**<0.001** ^e^
Female	124 (34^c^)	76 (30)	19 (33)	29 (59)	
Male	238 (66)	180 (70)	38 (67)	20 (41)	
Disease					0.31
Colon	200 (55)	136 (53)	36 (63)	28 (57)	
Colon and Rectum	43 (12)	36 (14)	4 (7)	3 (6)	
Rectum	119 (33)	84 (33)	17 (30)	18 (37)	
Previous chemo drug classes					0.98
No	349 (96)	247 (96)	55 (96)	47 (96)	
Yes	13 (4)	9 (4)	2 (4)	2 (4)	
Presence of liver metastasis					0.85
No	95 (26)	65 (25)	16 (28)	14 (29)	
Yes	267 (74)	191 (75)	41 (72)	35 (71)	
BMI					0.46 ^d^
Median (Min, Max)	26.5 (16.1, 49.5)	26.6 (16.6, 49.5)	25.0 (16.1, 48.9)	26.6 (17.3, 36.9)	
ECOG^d^					**0.001**
0	113 (31)	92 (36)	10 (18)	11 (22)	
1	212 (59)	145 (57)	34 (60)	33 (67)	
2	37 (10)	19 (7)	13 (23)	5 (10)	

^a^ *p*‐values for each variable with chi‐square test or *t*‐test for categorical and continuous data, respectively.

^b^ Min refers to the minimum value and Max refers to the maximum value in each cluster for continuous variables.

^c^ The number in parentheses represents the proportion (%) for each cluster.

^d^ ECOG, Eastern Cooperative Oncology Group.

^e^ *p*‐value less than 0.05 is bolded.

Table 3 shows the clinical variables in each GMM class in the control treatment arm. There were significant differences between the classes for sex and ECOG performance status ($p_{\mathrm{sex}}<0.001;p_{E\mathrm{COG}}<0.001$). Similar to the experimental treatment arm, Class 3C had more female than male CRC patients. Class 1C had the smallest number of CRC patients. All patients in Class 1C had ECOG performance status of 0 or 1, which meant that these patients were better functioning, when compared to the other classes.

**TABLE 3:** Differences in patient classes for clinical variables in the Cetuximab plus placebo treatment arm

Covariate	Full sample (*n* = 367)	Cluster 1C (*n* = 90)	Cluster 2C (*n* = 180)	Cluster 3C (*n* = 97)	*p*‐value^a^
Age					0.06
Median (Min, Max^b^)	63.4 (27, 87.9)	65.3 (28.1, 82.7)	63.4 (37.5, 87.9)	61.8 (27, 85.2)	
Sex					**<0.001** ^e^
Female	139 (38^c^)	27 (30)	50 (28)	62 (64)	
Male	228 (62)	63 (70)	130 (72)	35 (36)	
Disease					0.24
Colon	227 (62)	62 (69)	101 (56)	64 (66)	
Colon and Rectum	37 (10)	6 (7)	22 (12)	9 (9)	
Rectum	103 (28)	22 (24)	57 (32)	24 (25)	
Previous chemo drug classes					0.39
No	352 (96)	86 (96)	175 (97)	91 (94)	
Yes	15 (4)	4 (4)	5 (3)	6 (6)	
Presence of liver metastasis					0.45
No	110 (30)	23 (26)	54 (30)	33 (34)	
Yes	257 (70)	67 (74)	126 (70)	64 (66)	
BMI					0.38
Median (Min, Max)	26.5 (14.9, 46.3)	27.3 (16.3, 44.1)	26.5 (16.9, 46.3)	25.8 (14.9, 44.4)	
ECOG^d^					**<0.001**
0	123 (34)	51 (57)	52 (29)	20 (21)	
1	208 (57)	39 (43)	110 (61)	59 (61)	
2	36 (10)	0 (0)	18 (10)	18 (19)	

^a^ *p*‐values for each variable with chi‐square test or *t*‐test for categorical and continuous data, respectively.

^b^ Min refers to the minimum value and Max refers to the maximum value in each cluster for continuous variables.

^c^ The number in bracket is proportion (%) for each cluster.

^d^ ECOG, Eastern Cooperative Oncology Group.

^e^ *p*‐value less than 0.05 is bolded.

### 3.4 In each of the treatment arms, there are differences in overall survival (starting from 16 weeks onward) according to their classes that were identified by longitudinal global QoL scores (0–16 weeks)

**FIGURE 3:**
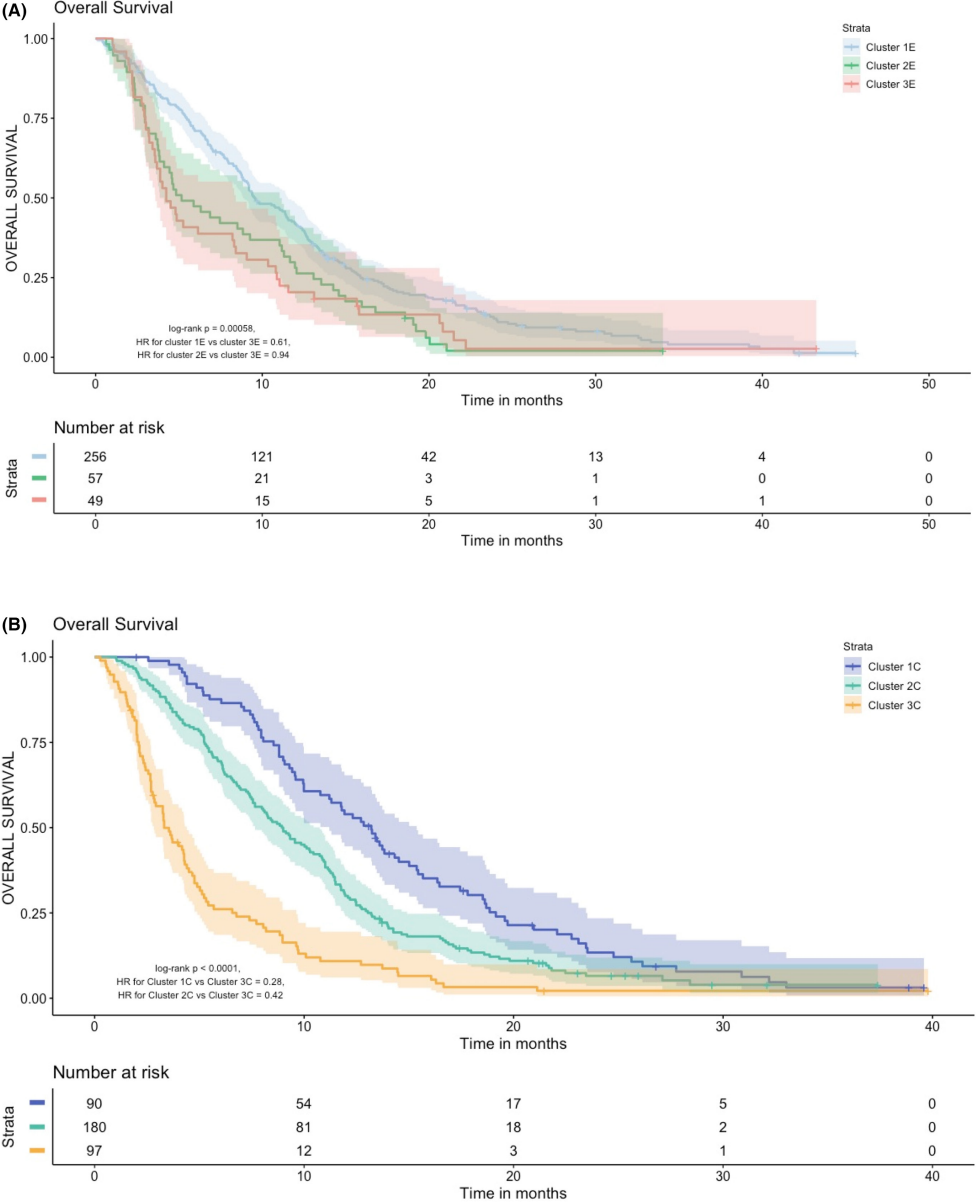
(A) Kaplan–Meier curves for overall survival for Cetuximab plus Brivanib treatment arm. 95% confidence interval is also shown with the shaded area. (B) Kaplan–Meier curves for overall survival for Cetuximab plus placebo treatment arm. 95% confidence interval is also shown with the shaded area

Figure 3A shows the OS for the three classes in the experimental treatment arm. The three QoL classes showed significantly different OS with the log‐rank p value = 0.0006. Class 3E had the worst survival. All CRC patients in Class 2E died within 35 months. Class 1E had most patients and the best survival. Class 3E with 49 patients had a convex curve. The survival of this cluster initially had the worst OS before 15 months, then performed better than Class 2E at 18 months. Class 1E with 256 patients had the highest QoL for all the time points and they had the longest OS.

Figure 3B shows the OS for the three classes in the control treatment arm. These classes showed significantly different OS patterns with the log rank *p* value <0.0001. As a class, Class 3C with 97 patients had the poorest OS. Class 1C with 90 patients had the longest OS. Patients with higher QoL had significantly better OS.

In addition, another sensitivity tests for OS were conducted for each of the two treatment arms with a shorter period. For this sensitivity analysis, we performed a landmark survival analysis using the first 8‐week period to generate the GMM patient classes, and then evaluated survival beyond 8 weeks, instead of our primary sensitivity analysis of using a 16‐week as cutoff (Figures S5 and S6). There were totally 52 patients who died within 8 weeks or had missing QoLs; and we excluded these patients in the landmark survival analysis. Our 8‐week sensitivity results for each of the two treatment arms showed consistent results to our 16‐week landmark analysis, indicating robust performance of the GMM model.

### 3.5 Overall Survival is associated with baseline, change slope of the QoL of individual patients

Table 4 shows the result of multivariable analysis on CRC survival using estimated individual QoL parameters on OS, among all CO.20 patients (regardless of treatment arm). The purpose was to evaluate each patient's QoL pattern over time. We allowed the intercept and slope of the QoL change for each arm to be different in this model. In this model, all the individual QoL parameters, the baseline, slope, and quadratic of the QoL from the GMM output had significant associations with OS (*p* < 0.001).

**TABLE 4:** Multivariable analysis on CRC survival using estimated individual QoL parameters

	HR^a^	*p* ^b^	95% CI^c^
Baseline	0.98	**<2e‐16** ^d^	(0.97, 0.98)
Slope	0.87	**<2e‐16**	(0.84, 0.9)
Quadratic	0.46	**4.58E‐15**	(0.38, 0.56)

^a^ HR, Hazard ratio.

^b^ p: *p*‐value of Cox regression analysis.

^c^ 95% CI: 95% confidence interval.

^d^ *p*‐value less than 0.05 is bolded.

## 4 DISCUSSION

In this study, we clustered patients separately for the experimental and control treatment arms of the CO.20 trial into several new patient classes through GMM. For the new patient classes or subgroups, each had a different longitudinal global QoL score trajectory that was associated with differences in their survival status and had different distributions by sex and performance status. Without GMM clustering, these differences might have been left undetected. When comparing the two treatment arms, there were no significant differences in baseline clinical characteristics and no differences in the outcome of OS between the two treatment arms; this provided a consistent baseline level for our analysis.

We hypothesized that different classes of CRC patients would present with different QoL longitudinal trajectories over time and that these differences might affect their OS. To test this hypothesis, we utilized GMM to cluster the patients of each treatment arm in separate analyses. We included variables in our GMM analyses that might affect the longitudinal QoL. Therefore, a LMM helped us to obtain the clinical variables that were strongly correlated with QoL: sex and ECOG.

Clustering is the unsupervised classification of objects (in this case, patients) into different groups.^27^ It segregates objects into different subgroups (which we termed, classes), such that patients in the same class are more similar or closer to each other and dissimilar to patients in other classes. Recently, GMM has become popular because GMM clusters individuals to different growth trajectory groups and characterizes the trajectories over time. These Growth trajectories are generated from a random effects algorithm that is also used in linear growth modeling. We can think of GMM as a combination of finite mixture analysis with conventional growth modeling. GMM is an extension of latent class growth analysis (LCGA),^26^ which allows the mean, variance, and covariance estimated for the growth factors to vary for each class. We can include predictors in the analysis to address putative confounding effects. GMM is increasingly applied in various fields, including psychology, education, public health, management, etc.^28^, ^29^, ^30^, ^31^ GMM was also applied in some recent studies which focused on cancer patients.^32^, ^33^, ^34^, ^35^ This study on longitudinal QoL of CRC patients clustered by GMM is among the first of this type have involved single time‐point QoL data. Moreover, single time‐point studies do not reveal the patterns of QoL change in patient subgroups over time. GMM modeling methods can track the trend of data development while considering individual heterogeneity; thus, repeated measurement data can be better clustered by GMM to find potential population differences.^36^

Our results demonstrated that GMM has satisfactory clustering ability for QoL data. After clustering CRC patients by their QoL data using GMM, each treatment arm was divided into three new classes. The proportion of each class in both treatment arms was larger than 10%. The entropies for the chosen models in the two treatment arms were higher than 0.6. Trajectories of estimated means of QoL for conditional GMM were significantly different for the classes of patients for the two treatment arms. To assess the association of QoL change and OS, we conducted landmark analysis by using the OS starting from 16 weeks and excluded the patients who died before 16 weeks. In addition to that, sensitivity tests were conducted by using the OS starting from 8 weeks and excluded the patients who died before 8 weeks. The results showed that there are still significant differences in survival curves among different classes, which further indicated that the stability of GMM was robust. Besides that, the identified subgroups based on the unsupervised learning show significant overall survival differences which infer the prognostic effect of longitudinal QoL measures during treatment.

Collectively in this study, GMMs can separate CRC patients well into different classes for each of the treatment arms. In our study, the discrimination ability of gender, ECOG and GMM classes, as shown by our reported C‐indices suggest that the selected variables could classify different groups well, which further verified the accuracy of our model.

We further compared the clinical characteristics of the new clusters and found that there were significant differences between gender and ECOG performance status in both the experimental and control treatment arms. In third classes (3E/3C) of each of the treatment arms, females had poorer QoL than males. A recent study showed that women reported more fatigue and pain than men for CRC patients.^37^ ECOG status was a predictor of adjuvant chemotherapy toxicity in patients with stage III colorectal cancer,^38^ which was similar to our study.

Finally, we found that the classes clustered by GMM are associated with differential OS, which is due to the different time‐related trajectories of QoL. Although clinicians understand that the higher the QoL, the better the OS, we need GMM to cluster the classes with potential heterogeneity. In addition, GMM outputted three parameters from the longitudinal QoL. They are all significantly correlated with survival performance.

## 5 LIMITATIONS

This study features some limitations. The first limitation is that the current samples are limited to CRC patients in Australia and Canada, which may influence our analysis and result. Future research is needed to validate the findings in other independent cohorts. Furthermore, we used 16 weeks of QoLs to predict overall survival beyond 16 weeks. We also considered using shorter time periods, for example, 8 weeks to predict survival performance. Both results are consistent. However, using 8 weeks follow‐up of QoL data provided reduced information for QoL change pattern estimation and results in higher variation. Finally, we acknowledge that in some studies, the QoL measures of patients might not be missing at random. We will extend our methodology to deal with other missing patterns in the future research.

## 6 CONCLUSION

GMM can be used as a clinical prediction and cluster tool to screen colorectal cancer patients with different potential survival states into different classes. GMM is designed for longitudinal analysis that can cluster patients into classes. GMM can effectively evaluate the longitudinal QoL characteristics of individuals, which can be used to predict survival performance of the patients and provide clinicians with some prognosis information for patients.

## AUTHOR CONTRIBUTIONS

**Jiahui Zhang:** Conceptualization (equal); formal analysis (equal); methodology (equal); writing – original draft (equal); writing – review and editing (equal). **Weili Kong:** Writing – original draft (equal); writing – review and editing (equal). **Pingzhao Hu:** Conceptualization (equal); formal analysis (equal); supervision (equal); writing – original draft (equal); writing – review and editing (equal). **Derek Jonker:** Data curation (equal); writing – review and editing (equal). **Malcolm Moore:** Data curation (equal); writing – review and editing (equal). **Jolie Ringash:** Data curation (equal); writing – review and editing (equal). **Jeremy Shapiro:** Data curation (equal); writing – review and editing (equal). **John Zalcberg:** Data curation (equal); writing – review and editing (equal). **John Simes:** Data curation (equal); writing – review and editing (equal). **Dongsheng Tu:** Data curation (equal); writing – review and editing (equal). **Chris J. O'Callaghan:** Data curation (equal); writing – review and editing (equal). **Geoffrey Liu:** Conceptualization (equal); supervision (equal); writing – review and editing (equal). **Wei Xu:** Conceptualization (equal); formal analysis (equal); methodology (equal); supervision (equal); writing – original draft (equal); writing – review and editing (equal).

## FUNDING INFORMATION

This study was supported by the Lusi Wong Fund, Princess Margaret Cancer Foundation, Alan Brown Chair in Molecular Genomics (to GL). WX was funded by the Natural Sciences and Engineering Research Council of Canada (NSERC Grant RGPIN‐2017‐06672).

## CONFLICT OF INTEREST

None.

## ETHICS STATEMENT

This study (registration number: NCT00079066) was approved by the National Cancer Institute of Canada (NCIC) Clinical Trials Group and the Australasian Gastro‐Intestinal Trials Group (AGITG). All participants provided written informed consent.

## Supporting information


Data S1

## Data Availability

The data that support the findings of this study are available on request from the corresponding author [WX, PH, JZ]. The data are not publicly available due to the containing information could have participant privacy. The source code is available at https://github.com/huiflora/stratify_crc.
