# Supplementary material for: Clustering on longitudinal quality‐of‐life measurements using growth mixture models for clinical prognosis: Implementation on CCTG/AGITG CO.20 trial
Source: Cancer Med. 2022 Oct 24;12(5):6117–28. doi: 10.1002/cam4.5341 (PMC10028035; doi:10.1002/cam4.5341)
Supplement: Supplementary file 1 — Data S1 [file CAM4-12-6117-s001.docx]

**Clustering on longitudinal quality-of-life measurements using growth mixture models for clinical prognosis: Implementation on CCTG/AGITG CO.20 trial**

Jiahui Zhang1 (jiahui.zhang@mail.utoronto.ca), Weili Kong2 (Weili.Kong@uhnresearch.ca), Pingzhao Hu1,3 (pingzhao.hu@utoronto.ca), Derek Jonker4 (djonker@ottawahospital.on.ca), Malcolm Moore5,6 (malcolm.moore@uhn.ca), Jolie Ringash7 (Jolie.Ringash@rmp.uhn.ca), Jeremy Shapiro8 (jeremy.shapiro@monash.edu), John Zalcberg9 (john.zalcberg@monash.edu), John Simes10 (john.simes@sydney.edu.au), Dongsheng Tu11 (dtu@ctg.queensu.ca), Chris J O'Callaghan 11 (cocallaghan@ctg.queensu.ca), Geoffrey Liu1,6,12* (geoffrey.liu@uhn.ca), Wei Xu1,13* (wei.xu@uhnresearch.ca)

1. **Supplementary Method**

**Description of the Growth mixture model**

Figure 1 shows the structure of conditional linear GMM. It can help to reveal population heterogeneity. By adding a covariate in the model, it can minimize potential confounding effect of subpopulation. The main model equations were as follows:

$$\begin{aligned} y_{itk}=\eta_{oik}+\lambda_{t}\eta_{1ik}+\varepsilon_{itk} \left( \text{1} \right) \end{aligned}$$

$$\begin{aligned} \eta_{oik}=\alpha_{0k}+\gamma_{ok}x_{ik}+\zeta_{oik} \left( \text{2} \right) \end{aligned}$$

$$\begin{aligned} \eta_{1ik}=\alpha_{1k}+\gamma_{1k}x_{ik}+\zeta_{1ik} (\text{3)} \end{aligned}$$

$$\begin{aligned} Pr\left( c_{ik}=1x_{ik} \right)=\frac{e^{\alpha_{ck}+\gamma_{ck}x_{ik}}}{\sum_{k=1}^{K} e^{\alpha_{ck}+\gamma_{ck}x_{ik}}},t=1,2,\cdots;i=1,2,\cdots n;k=1,2,\cdots K \left( \text{4} \right) \end{aligned}$$

where $\eta_{oik}$ and $\eta_{1ik}$ are latent intercept and slope terms for individual $i$ in class $k$; $x_{ik}$ is time-invariant covariate and $\lambda_{t}$ is the factor loading for slope at time $t$.$\alpha_{0k}$,$\gamma_{ok}, \alpha_{1k}$,$\gamma_{1k}$ are parameters.

**2. Supplementary Tables**

**Supplementary Table 1: Comparison of conditional GMMs with a quadratic term for the cetuximab plus brivanib treatment arm**

| Performance Criterion | Number of Classes^a^ | | | |
| --- | --- | --- | --- | --- |
|  | 2 | 3 | 4 | 5 |
| BIC^b^ | 14648 | **14669 ^e^** | 14669 | 14678 |
| Entropy^c^ | 0.54 | 0.75 | 0.8 | 0.75 |
|  |  |  |  |  |
| Proportion of each class ^d^ | 0.44 | 0.14 | 0.07 | 0.05 |
|  | 0.56 | 0.71 | 0.08 | 0.29 |
|  |  | 0.16 | 0.77 | 0.10 |
|  |  |  | 0.08 | 0.06 |
|  |  |  |  | 0.50 |

^a^ Number of classes for the GMM. ^b^ BIC, Bayesian information criterion. ^c^ Entropy for GMM. ^d^ Class proportion in column for classes for each GMM. ^e^ Smallest BIC value with entropy larger than 0.7, and each class has proportion larger than 10% .

**Supplementary Table 2: Comparison of the conditional GMMs with a quadratic term for cetuximab plus placebo treatment arm**

| Evaluation Criterion | Number of Classes^a^ | | | |
| --- | --- | --- | --- | --- |
|  | 2 | 3 | 4 | 5 |
| BIC^b^ | 14482 | **14312 ^e^** | 14459 | 14450 |
| Entropy^c^ | 0.78 | 0.69 | 0.83 | 0.84 |
|  |  |  |  |  |
| Proportion of each class ^d^ | 0.14 | 0.26 | 0.07 | 0.10 |
|  | 0.86 | 0.25 | 0.79 | 0.06 |
|  |  | 0.49 | 0.06 | 0.78 |
|  |  |  | 0.08 | 0.04 |
|  |  |  |  | 0.02 |

^a^ Number of classes for the GMM. ^b^ BIC, Bayesian information criterion. ^c^Entropy for the GMM. ^d^ Class proportion in column for classes for each GMM. ^e^ Smallest BIC value with entropy larger than 0.7, and each class has proportion larger than 10%.

**Supplementary Table 3:** **The discrimination ability of gender ,ECOG,** **Gender+ECOG, Gender+ECOG+Cluster with C-index**

| C-index | Gender | ECOG | Gender+ECOG | Gender+ECOG+Cluster |
| --- | --- | --- | --- | --- |
| Experimental arm | 0.51 | 0.61 | 0.62 | 0.63 |
| Control arm | 0.53 | 0.56 | 0.57 | 0.67 |

**3. Supplementary Figures**


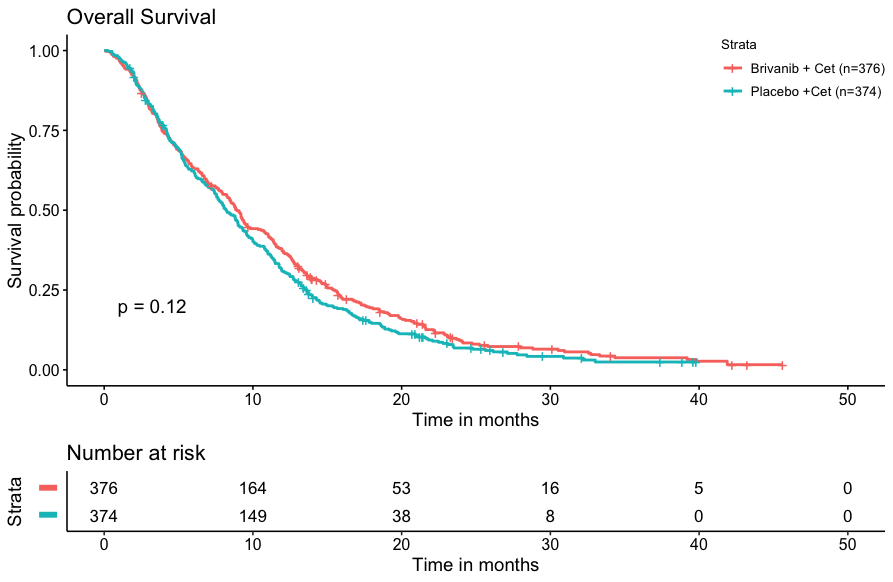
 **Supplementary Figure 1**: **Kaplan-Meier curve for overall survival in all randomized patients by treatment arm**


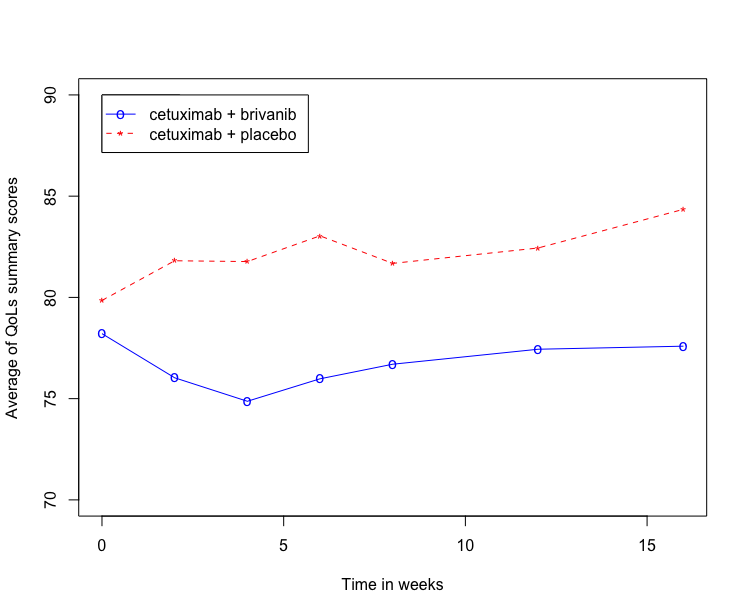


**Supplementary Figure 2**: **Mean of EORTC QLQ c30 summary scores for two treatment arms.**

Solid lines show the mean of QoL summary scores for the cetuximab plus brivanib treatment arm; dashed lines show mean of QoL summary scores for the cetuximab plus placebo treatment arm.


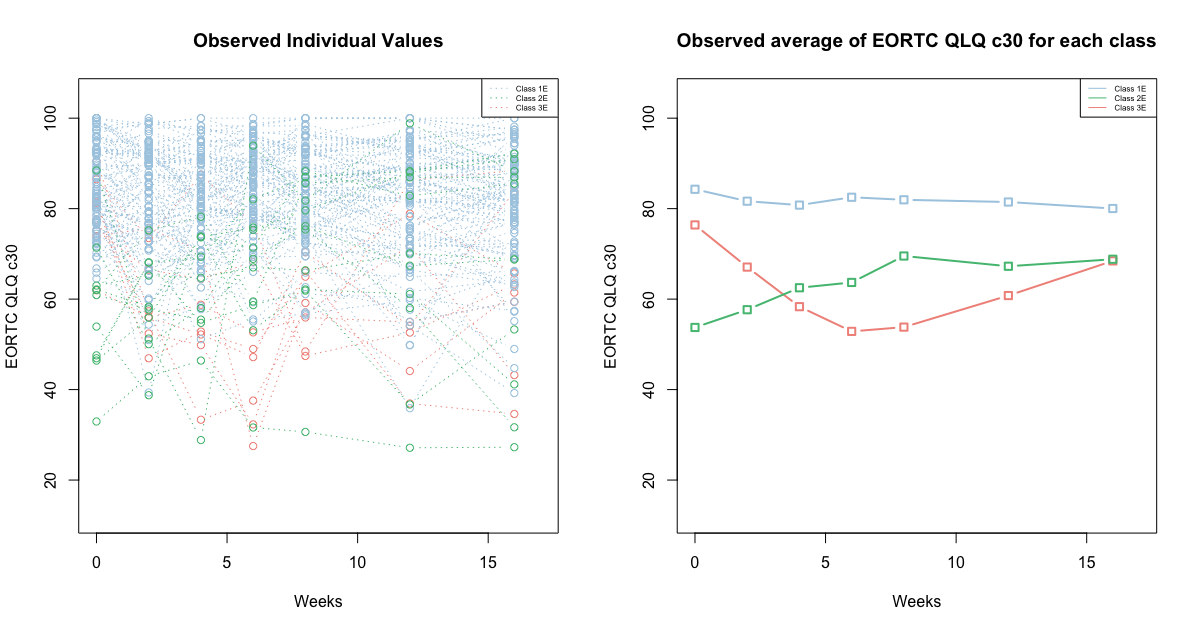


**Supplementary Figure 3**: **Observed Individual values and Observed average of individual values for 3-classes of conditional GMM for experimental arm**.

Solid lines represent observed mean values for each class; dashed lines represent observed individual values.


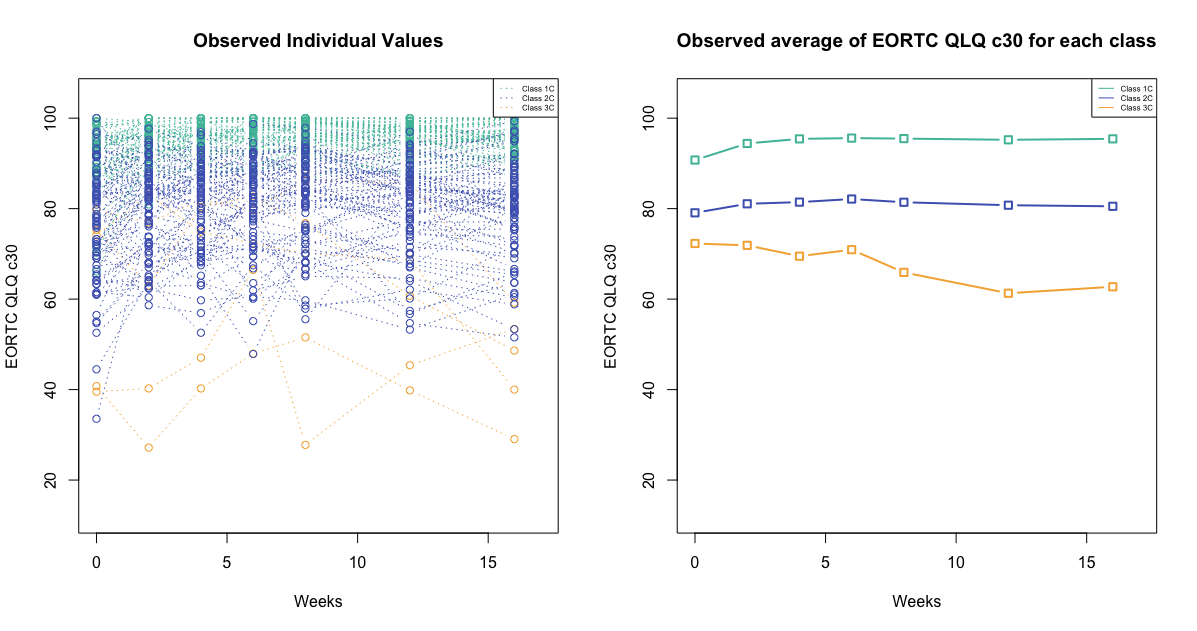


**Supplementary Figure 4**: **Observed Individual values and Observed average of individual values for 3-classes of conditional GMM for control arm**.

Solid lines represent observed mean values for each class; dashed lines represent observed individual values.


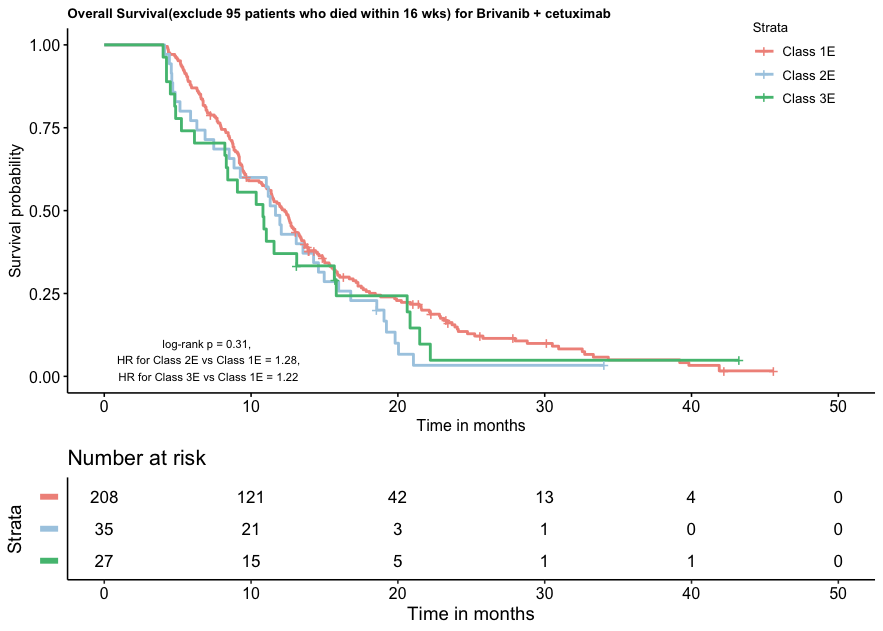


**Supplementary Figure 5: Sensitivity analysis for cetuximab plus brivanib treatment arm.**

Kaplan-Meier curves for overall survival when excluding patients who dies within 16 weeks.


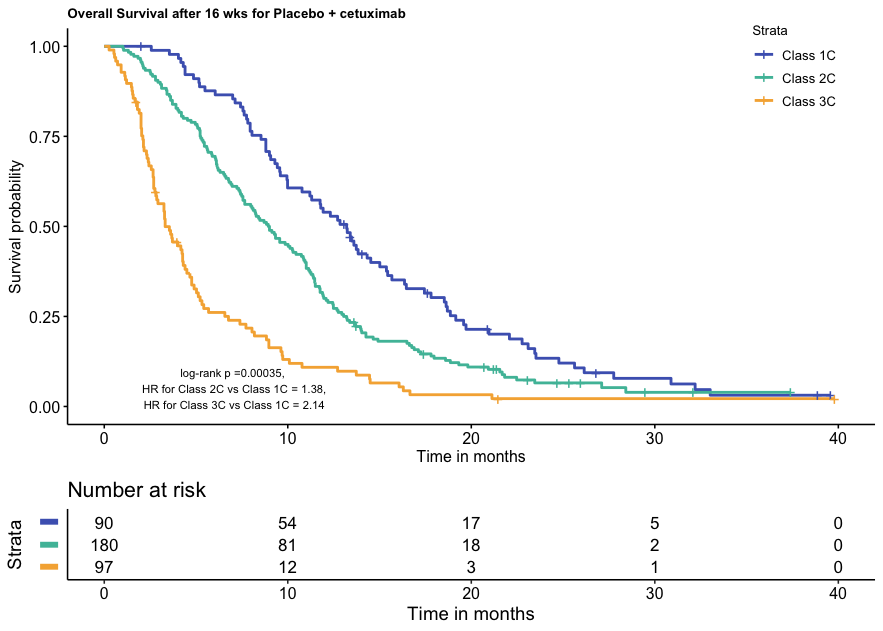


**Supplementary Figure 6: Sensitivity analysis for cetuximab plus placebo treatment arm.**

Kaplan-Meier curves for overall survival when excluding patients who dies within 16 weeks.
